# Close Males Sing With Dissimilar Minimum Frequency and Repertoire Size in a Wild Passerine

**DOI:** 10.1002/ece3.71044

**Published:** 2025-04-15

**Authors:** Mónika Jablonszky, Miklós Laczi, Gergely Nagy, Zoltán Tóth, Sándor Zsebők, László Zsolt Garamszegi

**Affiliations:** ^1^ Evolutionary Ecology Research Group Institute of Ecology and Botany, HUN‐REN Centre for Ecological Research Vácrátót Hungary; ^2^ Behavioural Ecology Group, Department of Systematic Zoology and Ecology ELTE Eötvös Loránd University Budapest Hungary; ^3^ HUN‐REN‐ELTE‐MTM Integrative Ecology Research Group Budapest Hungary; ^4^ Department of Zoology Plant Protection Institute, HUN‐REN Centre for Agricultural Research Budapest Hungary

**Keywords:** assortativity coefficient, bioacoustics, bird song, neighbor networks, within‐study meta‐analysis

## Abstract

The position occupied in social networks influences the success of individuals in many animal species. However, the associations between bird song (an important means of communication) and the relative position in social networks remained understudied. Such associations are expected because neighbors can learn song elements from each other or change their songs due to competition, and also because song can be related to other individual traits determining social network positions. We investigated these phenomena in males of the collared flycatcher ( *Ficedula albicollis* ), a passerine with complex songs and intense territorial interactions. Relying on 19 years of song recordings, we used multiple traits reflecting the spectral and temporal characteristics and complexity of songs, as well as syllable composition, to investigate if similarity in song is associated with the position in neighbor networks. We also examined whether birds settle down in an age‐dependent manner (as age is linked to individual quality) and whether the nonrandom spatial distribution of song is affected by the proportion of immigrants, young birds, or the number of displaying males. We found that the minimum frequency and the repertoire size of neighbors differed, but this pattern was not shaped by the investigated predictors. Therefore, our results highlight the need to study communication traits and social environment together. The fact that neighboring males tend to sing differently with respect to some song traits suggests that songs can be flexibly adjusted based on the performance of conspecifics.

## Graphical abstract

Spatial patterns in song can be influenced by social relationships, but this phenomenon is understudied. Using a long‐term dataset, we showed that close birds sing with different minimum frequencies and repertoire sizes, probably in order to make themselves distinguishable.

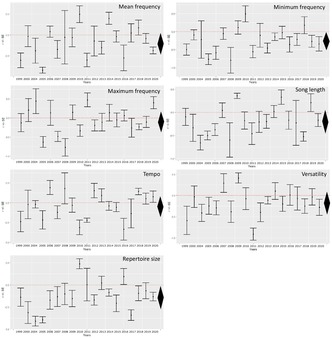

## 1 Introduction

In most animal species, individuals interact with other conspecifics throughout their lives nonrandomly, leading to the formation of social networks. The structure of social connections has been shown to influence mate choice and male–male competition in the population (Krause et al. 2007; Sih et al. 2009; Silk 2023). Individual traits like age (Smith et al. 2002; Aplin et al. 2015; Williams et al. 2023) or behavioral traits (Croft et al. 2009; Godfrey et al. 2012; Best et al. 2015; Williams et al. 2023) can determine the position of the individual within the network. Moreover, the network position and structure can also influence the animal's certain behavioral traits (Krause et al. 2010).

Social signals, such as bird songs, can play an essential role in mediating the interactions among individuals. For example, in territorial passerines with relatively stable social networks, where social network position is linked to spatial position (McDonald 2009; Sih et al. 2009; Silk 2023), songs can inform conspecific neighbors about the identity, quality, and motivation of the signaler (Cardoso et al. 2009; Rivera‐Gutierrez et al. 2010b; Warrington et al. 2014). However, songs can also be shaped by individual positions in the social network because, for instance, neighbors can influence each other's songs (Naguib et al. 2002; Liu 2004; Halupka 2014). Given the suspected influence of the social network on songs, these should be investigated together as a communication network to better understand the dynamics of vocal signaling. Furthermore, changes in such communication networks (such as reduced song sharing between neighbors) can be a sign of habitat deterioration (Laiolo and Tella 2005) caused by anthropogenic effects such as noise pollution (Nemeth and Brumm 2010; Grabarczyk and Gill 2020; Phillips et al. 2020), chemical pollution (Gorissen et al. 2005) or habitat fragmentation (Laiolo and Tella 2005; Rivera‐Gutierrez et al. 2010a; Pavlova et al. 2012).

Social networks can render spatial patterns in song characteristics among individuals to emerge on a small scale due to several nonmutually exclusive mechanisms. First, there may be assortative settlement based on traits reflecting quality, such as age (Latta and Faaborg 2002) or behavior, like aggression (Budka and Osiejuk 2017). Song can be associated with these traits, for example, age (Garamszegi et al. 2007; Bueno‐Enciso et al. 2016; Saldívar et al. 2020) or other behaviors (Linhart et al. 2013; Naguib et al. 2016; Szymkowiak and Kuczynski 2017), so nonrandom spatial distribution in song will also emerge (Sih et al. 2009; Krause et al. 2010; Aplin et al. 2015). Second, neighboring birds may learn from each other (Beecher et al. 1994; Koetz et al. 2007; Thomas et al. 2021) or match their songs to their neighbors (Foote et al. 2008; Price and Yuan 2011; Liu et al. 2018) resulting in similar songs in neighborhoods (Slater 1989). Singing similar songs in neighborhoods or regions was found to have various advantages for male birds, for example, in terms of female mate choice (Baker et al. 1987; Mortega et al. 2014) or lowering aggression from neighbors (Wilson and Vehrencamp 2001; Briefer et al. 2008; Draganoiu et al. 2014). Third, the song of birds singing close to each other may change due to competition. For example, song characteristics reflecting the quality of the singers increase if the density of males (that can be associated with the level of competition) is high (Williams and Slater 1990; Galeotti et al. 1997; Sexton et al. 2007). Furthermore, overall similarity in the spectral and temporal characteristics of the songs in neighbors can also increase with male competition (Laiolo and Tella 2005; Foote et al. 2008). Another possible effect of competition on the song of neighbors is that birds singing close to each other may change their song types (Vargas‐Castro 2015) or the frequency of their songs to remain audible (Mendes et al. 2011; Hamao et al. 2016) and facilitate recognition, causing negative assortativity among individuals in the frequency characteristics of the song.

Alternatively, spatial patterns in song characteristics can arise from mechanisms unrelated to the social network. For example, the songs of neighbors can be similar as the characteristics of their territory determining acoustic transmission are also similar, and the birds alter their songs accordingly (van Dongen and Mulder 2006; Goretskaia et al. 2018; Garcia et al. 2023). This scenario could lead to characteristic patterns, especially in the frequencies of songs, as high‐frequency sounds more rapidly degrade in dense foliage (Wiley 1991; Dabelsteen et al. 1993; Nemeth et al. 2001; Barker et al. 2009), but the degradation in temporal structure was also found to be associated with vegetation coverage (Wiley 1991; Nemeth et al. 2001; Bueno‐Enciso et al. 2016).

In this field study, we investigated the spatial distribution of characteristics related to the frequency, temporal structure, and complexity of the songs in neighboring collared flycatcher ( *Ficedula albicollis* ) males. Collared flycatchers are territorial passerines with complex and variable songs, and there is some evidence that it is an open‐ended learner species (Garamszegi et al. 2007; Zsebők et al. 2021; Vaskuti et al. 2022). However, the influence of neighbors on the song of this species was scarcely investigated, although we know that repertoire overlap is positively related to the distance between singing birds in this population on a scale of a few hundred meters (Garamszegi et al. 2012) and songs can be adjusted to the listening conspecifics (Jablonszky et al. 2021; Vaskuti et al. 2022). We created social networks based on the spatial position of singing territorial males to calculate similarity in song in relation to the position occupied in this network and formulated the following predictions (Table 1).

**TABLE 1:** Hypotheses and their predictions investigated in the study.

	Predictions for song traits (sign of assortativity)							Predictions for predictor variables (effect on the strength of assortativity)		
Hypothesis (mechanism of assortativity)	Mean F	Min F	Max F	SL	T	C	RS	Immigrants	1‐year‐old birds	Displaying males
Assortative settlement based on a quality indicator trait that is also related to song characteristics (e.g., age)	as age	as age					as age	−		
Learning from or matching songs to neighbors	+	+	+	+	+	+	+	−	−	
Competition	−	−	−	+		+	+	−	−	+
Transmission similarities of adjacent sites	+	+	+	+	+			−		

*Note:* Song traits used: Mean frequency (Mean F), minimum frequency (Min F), maximum frequency (Max F), song length (SL), tempo (T), complexity (C), and repertoire size (RS). Predictor variables used: Proportion of immigrants, proportion of 1‐year‐old birds, and average number of tested displaying males. Missing values indicate that we have no explicit prediction for that association.

If nonrandom spatial distribution in song emerges due to assortative settlement based on a quality indicator trait that is also related to song characteristics, we predicted similar spatial patterns in certain song traits (such as mean and minimum frequency and repertoire size) and age which were found to be linked in our population (Garamszegi et al. 2007; Jablonszky et al. 2021; Zsebők et al. 2021). If birds learn from or match their songs to their neighbors, we predicted that neighbors would use similar song elements in a similar way, leading to positive assortative patterns in all investigated song traits. Under this scenario, we can also expect changes in the pattern of similarity with the proportion of 1‐year‐old birds in the population, as they may be worse learners due to their inexperience (Ortega et al. 2014). If the song of neighboring males is rather influenced by competition, we predicted positive assortment in the song traits signaling quality, such as song length, complexity, and repertoire size (Garamszegi et al. 2006a; Garamszegi et al. 2007; Hegyi et al. 2010), and negative assortment in frequency measures (see above). In this case, we also expected that these patterns would change according to the number of other males displaying courtship behavior and/or the proportion of young birds, as these variables could reflect some aspects of the competition strength perceived by the focal males. If the transmission similarities of adjacent sites govern the nonrandom spatial distribution of song characteristics, we predicted a positive assortment in frequency measures, tempo, and song length. All of these patterns can be somewhat influenced by the proportion of immigrants arriving in the population that may bring very different songs and may have difficulties adjusting to local songs and local settlement patterns (Fayet et al. 2014; Parker et al. 2022; Barta et al. 2023). Thus, we also investigated the effect of the proportion of immigrant birds on the similarity of the songs of neighbors. Additionally, we examined the spatial pattern in repertoire similarity to check whether changes in the used repertoire elements drive the potential nonrandom spatial distribution of the song traits.

## 2 Methods

### 2.1 Fieldwork

The study area consisted of nest box plots situated in an oak‐dominated forest area managed by the Duna‐Ipoly National Park, Hungary (47°43'N, 19°01′ E). There were 400–600 artificial nest boxes in the area in the study years (1999–2020). The nest boxes were arranged in a grid system, with an average distance of around 31 m from each other Recordings were made at nest boxes at most 2 km from each other.

The study species was the collared flycatcher, a cavity‐nesting, long‐distance migratory passerine with a large individual syllable repertoire size (20–200 syllables per 20 songs) and flexible song structure (Garamszegi et al. 2006a; Zsebők et al. 2018b; Zsebők et al. 2021). Although within‐day repeatability was found to be significant in every studied song trait, it was < 0.25 for mean frequency, frequency bandwidth, song length, tempo, and complexity, and higher only for repertoire size and song rate. Among‐day and among‐year repeatability was not significant, except for complexity (0.2) in the among‐day context (Zsebők et al. 2017). Song traits are shaped by internal state (Garamszegi et al. 2004), age (Garamszegi et al. 2007; Jablonszky et al. 2021; Zsebők et al. 2021) and can be plastically adjusted to a degree to the identity of the listening conspecifics and song post height (Jablonszky et al. 2021; Jablonszky et al. 2022b).

Male collared flycatchers usually arrive in the middle of April at the study site, then establish a small territory (including the nest box only or the immediate surroundings of the nest box) and start to sing. The most active period of singing is in the second half of April and the beginning of May when males fight for and occupy territories and court females. In this period, males can still change territories frequently, and birds generally stop singing after pairing, so males usually sing for 2–7 days in the same nest box (Garamszegi et al. 2012).

The recordings for this study were made in 1999, 2000, and 2004–2020 between April 11 and May 9 and include data from 421 birds. A total of 8–45 individuals (mean ± SD: 22.16 ± 11.08, see also Table S1) were included in the analysis yearly, which corresponded to approximately 18.61% ± 11.04% of the breeding collared flycatcher males. We used only one recording from an individual per year, and the number of birds with two recordings from different years was low (*N* = 12) which could induce only minor bias in the analysis (if we exclude these birds the main findings of the study remain qualitatively unchanged). Unfortunately, the among‐year sample size did not allow us to further explore the within‐individual changes between years and the effect of returning individuals on song organization. A detailed procedure for song recording can be found elsewhere (Garamszegi et al. 2006a; Zsebők et al. 2018b). Shortly, we searched in the study area daily for singing birds showing courtship display around a nest box and recorded their song with a parabola microphone and digital recorders. Recordings were made in good weather conditions (without strong wind and rain), lasted at least 10 min, and included at least 20 songs. Recordings were stopped when major disturbances from conspecifics or other animals occurred.

We captured the focal males after song recordings for ringing and to perform morphological measurements. Birds without rings were marked with individually numbered rings (Aranea, Poland, official rings of the Hungarian Bird Ringing Centre). We determined the age of males (i.e., 1‐year old or older) based on their plumage (Mullarney et al. 1999).

We used variables in the statistical analyses (see below) describing the social environment and demographic conditions during the courtship period of the study species (when song recordings were made). These variables were calculated as follows. In the courtship period, we also conducted behavioral tests connected to nest display and territorial aggression apart from song recordings in 2004, 2005, and 2007–2020 (see, e.g., Garamszegi et al. 2006b; Garamszegi et al. 2009; Krenhardt et al. 2024). The behavioral variables obtained during these tests were not associated with song traits (Garamszegi et al. 2008). As these behavioral tests were connected to the courtship behavior of the males and we daily monitored the study area for displaying birds, we could assess a variable related to the social environment, namely, the average number of tested displaying males (mean daily number of males showing courtship behavior with behavioral test), and demographic parameters, namely the proportion of 1‐year‐old males and the proportion of immigrants (proportion of birds captured without ring) during courtship for the years. These parameters are estimated with some noise. Weather conditions and the capacity of the research group can somewhat influence sampling during the courtship period. However, as behavioral tests and song recordings were conducted on the same days and in the same area, the number of behavioral tests can reliably estimate the other males in the vicinity with which the focal males have to compete for territories and females. As not all tested birds sang enough for recording, the number of tests reflects better the number of displaying males than the number of recordings. Furthermore, birds without rings are not necessarily immigrants; some could have been born in a natural nest hole in the study area. However, due to our thorough sampling effort, we believe that these sources of noise cause only minor biases in our results.

We followed all applicable international, national, and/or institutional guidelines for animal care and use. Field protocols were approved by the ethical committee of the ELTE Eötvös Loránd University (ref. no. TTK/2203/3). Behavioral assays, captures, and measurements were undertaken as cautiously and efficiently as possible, in a way that minimizes welfare impacts on the birds. Permissions for the fieldwork have been provided by the Government Office of Pest County National Inspectorate for Environment and Nature, reference numbers: DINP 2256–3/2002, DINP 1931–2/2003, DINP 2573/2/2004, KTVF/15951/2005, KTVF/22021/2006, KTVF 16360–2/2007, KTVF 30871–1/2008, KTVF 43355–1/2008, KTVF 45116–2/2011, KTVF 21664–3/2011, KTVF 12677–4/2012, KTVF 10949–8/2013, KTF 11978–5/2015, PEI/001/1053–6/2015, PE/EA/101–8/2018, PE‐06/KTF/8550–4/2018, and PE‐06/KTF/8550–5/2018.

### 2.2 Song Analysis

Seven independent song traits with suspected biological relevance in the study population and related to one or multiple hypotheses detailed in the introduction were used during the statistical analysis (Zsebők et al. 2017; Jablonszky et al. 2021). Specifically, we considered for each recording the mean, minimum, and maximum frequency, as well as song length, tempo, complexity, and repertoire size.

As a first step, we manually cut out the songs of sufficient quality from the recordings using Adobe Audition 3.0 (Adobe Systems) software. Then, the Ficedula Toolbox (Zsebők et al. 2018a) was used to manually segment the syllables from the songs by selecting the start and end points of the syllables. With the same program, we extracted the mean frequency for each syllable automatically. Thus, we avoided potential bias arising due to the manual assessment of frequency measures (Brumm et al. 2017). Also, with the Ficedula Toolbox, we manually clustered the syllables into syllable types at the individual level and then made clustering at the among‐individual level (see Zsebők et al. 2018a for further details). The clustering was based on the visual similarity of the syllables (e.g., their frequency, shape, and length) and was aided by the Ficedula toolbox that provides an interface where the uncategorized syllables can be seen ordered according to their acoustic similarity and syllable type categories are also suggested. Thus, the researchers could more easily categorize similar syllables into syllable types. The among‐individual clustering was conducted on the syllable types identified at the within‐individual level. The manual part of these processes was carried out by multiple trained researchers following a strict protocol and involving regular checks, so the bias by different researchers should be minimal, as was confirmed by a previous study (Zsebők et al. 2018a). Examples of syllable types with multiple syllables from multiple individuals (to show within‐ and among‐individual variance in syllable types) can be seen in Figure S1 in the Supporting Information.

At the level of songs, we calculated the song length and tempo (the number of syllables within the song divided by song length, 1/s). Complexity was computed as the number of different syllable types divided by the total number of syllables in each song. Additionally, we calculated the mean, minimum, and maximum frequencies for each song based on the mean frequencies of the syllables of the song. Then, we averaged the measures of 15 songs from each male to obtain one value per individual. Repertoire size was the number of different syllable types in 15 songs. Summary statistics of the song traits are presented in Table S2.

Repertoire similarity between the recordings was calculated for the first 14 years (from which syllable clustering at the among‐individual level was available) as a Jaccard index based on the syllable categories (the number of the common syllable types of two birds was divided by the total number of their syllable types).

### 2.3 Statistical Analyses

To study the spatial distribution of song characteristics among neighboring singing collared flycatchers and the potential mechanism of this phenomenon, we investigated the strength and direction of similarity of bird song in neighbor networks (as yearly weighted assortativity coefficients), the dependence of these spatial patterns on social, environmental, and demographic variables (average number of tested displaying males, proportion of young birds and proportion of immigrants) and their relationship with settlement patterns in age.

#### 2.3.1 Software Used

All analysis was conducted in the R 4.1.2 programming environment (R Core Team 2019). We calculated autocorrelation with the “acf” function from the “stats 4.3.2” package. We carried out Voronoi/Dirichlet transformation using the “deldir” function from the “deldir 2.0–0” package (Turner 2023). We used the “assortment. Discrete” and “assortment. Continuous” functions from the “assortnet 0.20” package (Farine 2023) to calculate assortativity coefficients and “mantel” function from the “vegan 2.6–4” package (Oksanen et al. 2020). Figures were created with the help of the “deldir 2.0–0” (Turner 2023), “igraph 1.6.0” (Csardi and Nepusz 2006), and “ggplot2 3.4.4” (Wickham 2016) packages.

#### 2.3.2 Network Construction and Calculation of Assortativity

**FIGURE 1:**
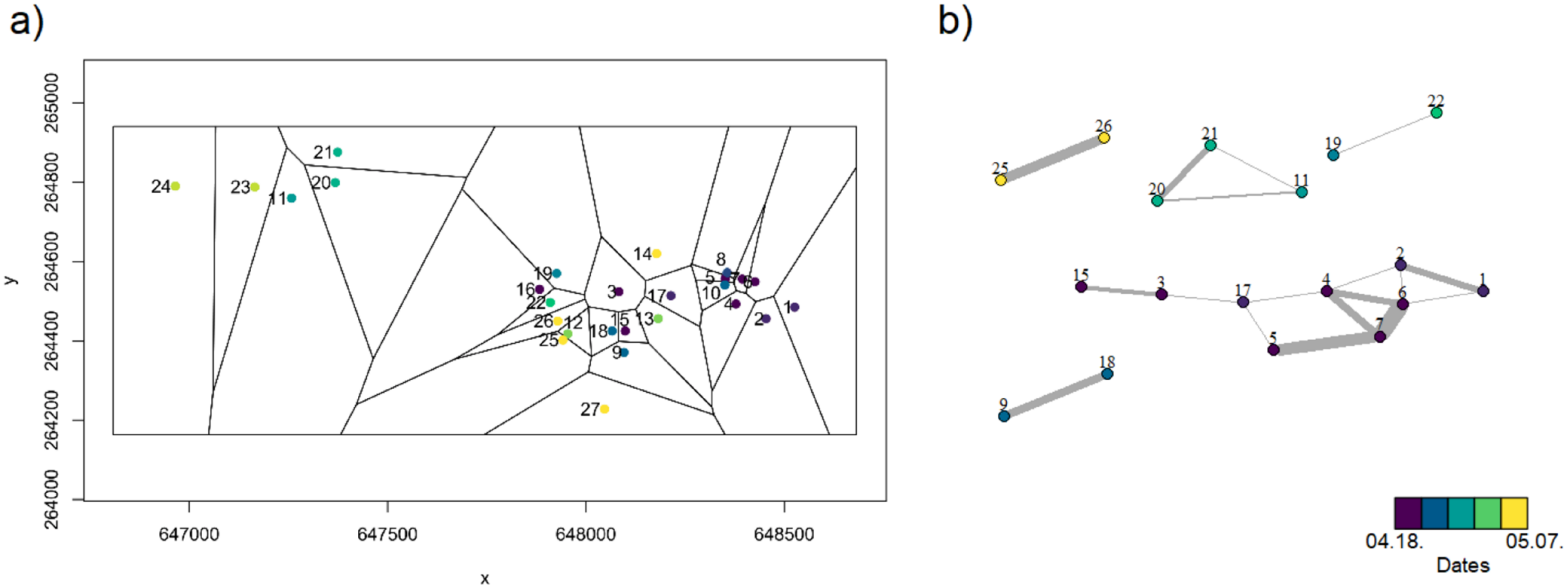
Depiction of estimated areas where birds could interact based on Voronoi/Dirichlet tessellation (a) with *x*‐ and *y*‐coordinates on the axes and the final neighbor networks (b) of a typical year (2014) with an average sample size (*N* = 27). Numbers indicate individual males and colors depict recording dates according to the scale in the figure. In (a), the points represent nest boxes where song recordings were made. In (b), the points represent males and the lines between them indicate neighbor relationships (birds that could hear and interact with each other) weighted by the spatial and temporal distance between them. Birds were regarded as neighbors only if their nest boxes were within 200 m of each other and they sang within 3 days.

We calculated yearly weighted assortativity coefficients (*r*; Newman 2002; Farine 2014) for every song trait (range: −1 to 1) to measure whether associations in the neighbor networks are stronger between phenotypically similar (values closer to 1) or dissimilar individuals (values closer to −1). For this, we constructed one neighbor network for each year by using the Voronoi/Dirichlet tessellation method (Lee and Schachter 1980) on the spatial position of the occupied nest boxes to define Voronoi cells of which points are closer to the focal nest box than to any other nest boxes, and we classified the singing males as potential neighbors if they shared a section of the Voronoi cell border. The networks included 8–45 individuals per year (see also above). Further, we regarded birds as neighbors only if they could hear each other and interact vocally with each other. We applied two thresholds to assure that the birds had the chance to vocally interact: the nest boxes of the neighbors were a maximum of 200 m from each other, and their songs were recorded within 3 days (Figure 1). The distance threshold was determined based on previous studies of forest birds (Dabelsteen et al. 1993; Naguib et al. 2008) and based on our 20 years of field experience with the species. The limit of 3 days was determined given that males usually sang for only 2–7 days (personal observation of the authors, see also: Garamszegi et al. 2012). Including higher day and distance limits (that may be applied if one considers that birds can fly further away from their nest boxes to find food or water) did not influence the results qualitatively. Nevertheless, we used the lowest considered values to avoid including birds that could not interact. For calculating the weighted assortativity coefficients with their standard errors each year, we weighted the edges in the neighbor networks by the actual spatial and temporal distance between the birds (Farine 2014). The weighting was done by multiplying neighbor status (0 or 1) with the inverse of distance and days between recordings (more than 200 m and more than 3 days were turned to 0).

Because the repeatability of song characteristics is low and we could not consider the within‐individual variance during the calculation of weighted assortativity coefficients, we carried out additional analyses determining within‐recording repeatability and assessing the effect of within‐individual variation on the results by repeating the analyses with randomly chosen songs instead of their mean to validate our main results (see Supporting Information). These additional analyses did not include repertoire size because it is an inherently individual‐specific trait that is estimated without within‐individual variance. The additional analyses gave qualitatively similar results, but within‐individual variation added a higher level of uncertainty to the estimates that should be considered when interpreting our results.

#### 2.3.3 Autocorrelations in and Correlations Among the Yearly Assortativity Coefficients

We checked independence among the yearly assortativity coefficients by calculating autocorrelation and its confidence interval for every song trait for the consecutive years (with time lag of 1 for the period between 2004 and 2020). Significant positive autocorrelation would mean that the estimates of consecutive years are similar, thus nonindependent, which could arise due to cultural drift or because of environmental similarities between close years. To assess whether assortativity follows similar patterns among the years in different song traits, we also calculated Spearman's rank correlation between the yearly assortativity coefficients for each pair of song traits. We calculated 95% confidence intervals for the correlation coefficients using a method described by Bonett and Wright (2000).

#### 2.3.4 Average Effect Sizes and the Effect of Moderator Variables for the Song Traits

To investigate the predictions of the three latter hypotheses in Table 1 that predicted different assortativity patterns for different song traits and different effects of social environmental and demographic variables (hereafter moderator variables according to the meta‐analysis literature), we conducted within‐study meta‐analysis to calculate the average effect size for the weighted assortativity coefficients and assess the effect of the moderator variables. We conducted within‐study meta‐analyses for every song trait by considering that the calculated yearly assortativity coefficients are standardized effect sizes along the −1 to 1 scale similar to correlation effect sizes. Thus, values between 0.1 and 0.3 represent small, 0.3–0.5 medium, and > 0.5 high effect sizes (Cohen 1988). To investigate the overall effect and the heterogeneity among yearly assortativity estimates, we used Bayesian models from the MCMCglmm package (Hadfield 2010). We included the yearly assortativity coefficients as response variables and their standard errors as measurement error variance. As the environmental conditions can be similar in closer years, we controlled for this nonindependence by including year as a random factor connected to the inverse of a correlation matrix between the years in all models (Nakagawa and Santos 2012). The correlation matrix considered to reflect the expected among‐year similarity contained correlations that became weaker between more distant years, reaching 0 in years, 6 years apart. We used inverse gamma priors for the random variable. We ran the model for 510,000 iterations, with a thinning interval of 500, and we discarded 10,000 samples at the beginning. We checked the shape and trace of the posterior distributions, the autocorrelation between iterations, and examined potential mixing and convergence problems using the Gelman–Rubin statistics (Gelman and Rubin 1992). Using parameter‐expanded priors did not influence our results (not shown). The average effect size corresponded to the intercept of these models. We calculated heterogeneity (*I* ^*2*^) by dividing the among‐year variance by the sum of all variance components and a term based on the measurement error variance (Higgins and Thompson 2002; Nakagawa and Santos 2012). *I* ^*2*^ = 25% is considered low, *I* ^*2*^ = 50% moderate, and *I* ^*2*^ = 75% high heterogeneity (Higgins et al. 2003).

Next, we evaluated the influence of social, environmental, and demographic variables (namely, the average number of tested displaying males, the proportion of immigrant birds, and the proportion of 1‐year‐old birds during the courtship period) on the yearly assortativity estimates for every song trait. These variables could be calculated for only 16 years (see above); otherwise, the sample was the same as above. These variables seem to be independent based on Spearman's rank correlation (average number of tested displaying males and proportion of immigrants: *r*_*sp*_ = 0.362, *p* = 0.153; average number of tested displaying males and proportion of 1‐year‐old birds: *r*_*sp*_ = −0.297, *p* = 0.247; proportion of immigrants and proportion of 1‐year‐old birds: *r*_*sp*_ = 0.179, *p* = 0.491). Additionally, the investigated variables varied substantially during the study years [average number of tested displaying males: mean ± SE: 2.951 ± 0.834 (range: 1.667–4.000); proportion of immigrants: 0.387 ± 0.180 (0.125–0.727); and proportion of 1‐year‐old birds: 0.419 ± 0.252 (0.053–1.000)]. Due to our limited sample size (*N* = 16 years), we included these variables as explanatory variables one by one in the MCMCglmm models.

#### 2.3.5 Repertoire Similarity and Spatial Distance

Additionally, we checked whether there was a spatial pattern in song content by comparing repertoire similarity and spatial proximity matrices with Mantel tests. We did not calculate assortativity coefficients here but used Mantel tests because repertoire similarity is conveniently modeled as a matrix, and the latter method is ideal for matrix comparisons. We calculated Mantel correlations for the first 14 years for which data on repertoire similarity were available. The sample size was otherwise the same as for the analysis of weighted assortativity coefficients. Here, we also excluded birds that were recorded more than 200 m and more than 3 days apart. We calculated 95% confidence intervals for the yearly Mantel correlation estimates using the variance obtained for the estimates transformed to Fisher's *z* with the “escalc” function from the “metafor” package (Viechtbauer 2010). Then we analyzed the yearly Mantel correlations similarly to those above, including yearly estimates as a response variable to the MCMCglmm model, but calculating measurement error from the yearly sample sizes (1/(*N*‐3)).

#### 2.3.6 Relationships Between the Assortativity Coefficients of Song Traits and Age

To investigate the predictions of the hypothesis that age‐dependent settlement drives nonrandom spatial distribution in song, we calculated assortativity coefficients for binary age (1‐year‐old or older) and applied a meta‐analytic approach to examine if there was an overall tendency among the yearly assortativity coefficients in the same way as for the song traits. If we found such a pattern, we would compare the yearly assortativity coefficients of binary age and of song traits previously found to be associated with binary age: mean, minimum frequency, and repertoire size (Garamszegi et al. 2007; Jablonszky et al. 2021). To do that, we would calculate Spearman's rank correlations between the yearly assortativity coefficients of the song traits and binary age to investigate whether the strength and direction of association in neighbor networks are similar for age and the song traits. Data on binary age were missing for some individuals, preventing us from calculating weighted assortativity coefficients, so the sample size for this analysis became 14 years (see details in Table S1).

## 3 Results

### 3.1 Autocorrelations and Correlations Among the Yearly Assortativity Coefficients

We did not find strong autocorrelation (association between the values of subsequent years) among the yearly assortativity coefficients in either song trait. The autocorrelation values were −0.187 for mean frequency, −0.145 for minimum frequency, −0.307 for maximum frequency, −0.097 for song length, 0.002 for tempo, −0.088 for complexity, and 0.098 for repertoire size, all of which were within the 0.475 and −0.475 confidence interval calculated by the autocorrelation function, meaning that autocorrelations could not be differentiated from chance. We found only three significant correlations between the yearly assortativity coefficients of song traits, but generally, they were weakly correlated (see Table 2).

**TABLE 2:** Spearman's rank correlation between the yearly assortativity coefficients for the song traits with 95% confidence intervals.

	Mean frequency	Minimum frequency	Maximum frequency	Song length	Tempo	Complexity
Minimum frequency	**0.542** (0.082, 0.812)					
Maximum frequency	−0.126 (−0.550, 0.350)	0.004 (−0.451, 0.457)				
Song length	0.056 (−0.409, 0.498)	0.396 (−0.089, 0.730)	0.098 (−0.374, 0.530)			
Tempo	0.304 (−0.186, 0.672)	−0.105 (−0.535, 0.368)	0.035 (−0.426, 0.482)	−0.284 (−0.660, 0.205)		
Complexity	0.346 (−0.143, 0.699)	**0.489** (0.017, 0.783)	−0.077 (−0.514, 0.391)	**0.586** (0.140, 0.834)	−0.232 (−0.625, 0.255)	
Repertoire size	0.104 (−0.369, 0.534)	0.035 (−0.426, 0.482)	−0.065 (−0.505, 0.402)	0.240 (−0.247, 0.630)	−0.174 (−0.584, 0.308)	0.181 (−0.302, 0.589)

*Note:* Values are in bold when their 95% confidence intervals exclude zero.

### 3.2 Average Effect Sizes and the Effect of Moderator Variables for the Song Traits

**FIGURE 2:**
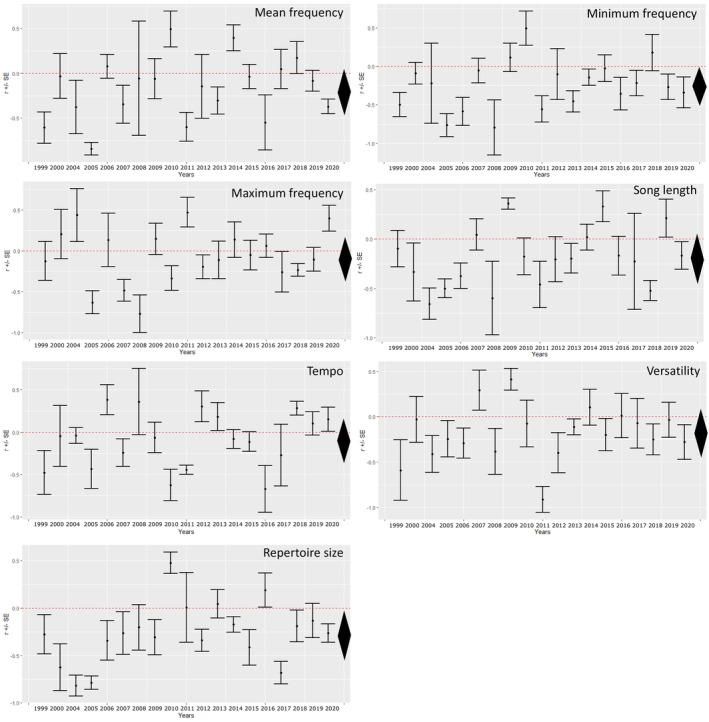
Yearly assortativity coefficient estimates (*r*) for all investigated song traits with 95% credible intervals. The average effect size and its 95% credible interval are depicted as a diamond on the right side for each song trait.

We found that two estimates for the average effect size of the yearly assortativity coefficients, of minimum frequency and repertoire size, were significantly smaller than 0 when considering their credible intervals (Figure 2 and Table 3). Although the effects were small, the negative value means that neighboring birds overall sang more dissimilar songs. Note that all estimates were negative. We found low but significant heterogeneity among the yearly estimates regarding all song traits, implying that the strength and direction of the association between the birds differed over the years.

**TABLE 3:** Average effect sizes (regression coefficient for the intercept) and heterogeneity of yearly assortativity coefficients of the investigated song traits.

Song trait	Average effect size (95% CI)	Heterogeneity (I^2^, 95% CI)	Average number of tested displaying males (95% CI)	Proportion of immigrants (95% CI)	Proportion of 1‐year‐old birds (95% CI)
Mean frequency	−0.214 (−0.485, 0.061)	28.165 (1.521, 64.494)	0.003 (−0.282, 0.295)	0.163 (−0.116, 0.411)	0.093 (−0.207, 0.396)
Minimum frequency	**−0.248** (−0.513, −0.009)	25.391 (2.218, 60.445)	−0.004 (−0.272, 0.274)	0.082 (−0.163, 0.327)	−0.077 (−0.356, 0.203)
Maximum frequency	−0.099 (−0.359, 0.165)	27.217 (3.143, 62.327)	0.031 (−0.223, 0.318)	0.113 (−0.131, 0.399)	−0.008 (−0.342, 0.191)
Song length	−0.174 (−0.425, 0.063)	28.338 (2.286, 63.705)	0.020 (−0.280, 0.287)	0.193 (−0.054, 0.433)	−0.037 (−0.288, 0.266)
Tempo	−0.081 (−0.338, 0.146)	26.591 (2.560, 58.597)	−0.051 (−0.282, 0.212)	−0.010 (−0.232, 0.279)	0.191 (−0.059, 0.412)
Complexity	−0.176 (−0.470, 0.086)	26.856 (2.464, 62.158)	−0.099 (−0.350, 0.196)	0.111 (−0.133, 0.388)	0.042 (−0.223, 0.311)
Repertoire size	**−0.294** (−0.555, −0.035)	30.939 (2.374, 65.690)	0.087 (−0.156, 0.352)	0.144 (−0.108, 0.383)	0.002 (−0.255, 0.268)

*Note:* The regression coefficients of the three moderator variables, the average number of tested displaying males, the proportion of immigrants, and the proportion of 1‐year‐old birds, are also shown. Estimates are presented with their 95% credible intervals (CI), and average effect sizes are in bold if their 95% CI does not include zero.

We found no effect of the moderator variables reflecting social, environmental, and demographic characteristics in any of the models (see Table 3).

### 3.3 Repertoire Similarity and Spatial Distance

According to the Mantel tests, there was no relationship between repertoire similarity and spatial distance in most of the years (Mantel *r* between −0.397 and 0.191, *p* = 0.170–0.991), except in 2011 (Mantel *r* = 0.397, *p* = 0.005; see Figure 4). The overall effect size was small and nonsignificant (−0.013, 95% CI: −0.233‐0.194).

### 3.4 Relationships Between the Assortativity Coefficients of Song Traits and Age

**FIGURE 3:**
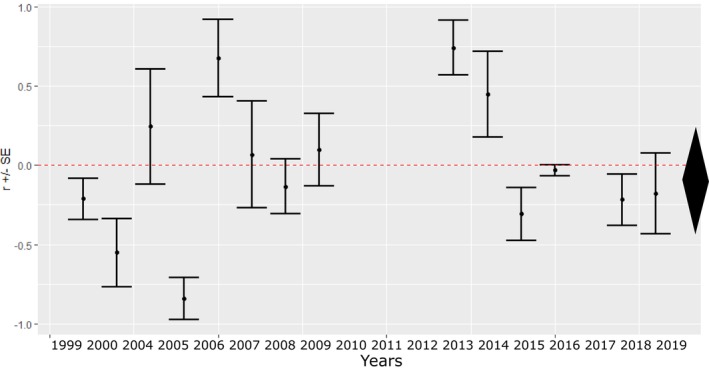
Yearly assortativity coefficient estimates (*r*) for binary age (1‐year‐old or older) with 95% credible intervals. The average effect size and its 95% credible interval are depicted as a diamond on the right side of the figure.

There was no clear pattern among the yearly assortativity estimates regarding binary age (estimate for average effect size: −0.058, 95% credible interval: −0.438, 0.247; Figure 3). Thus, we did not investigate the relationship between the assortativity coefficients of song traits and binary age. There was significant heterogeneity among the years (*I* ^*2*^: 33.732, 95% credible interval: 1.802–75.141).

**FIGURE 4:**
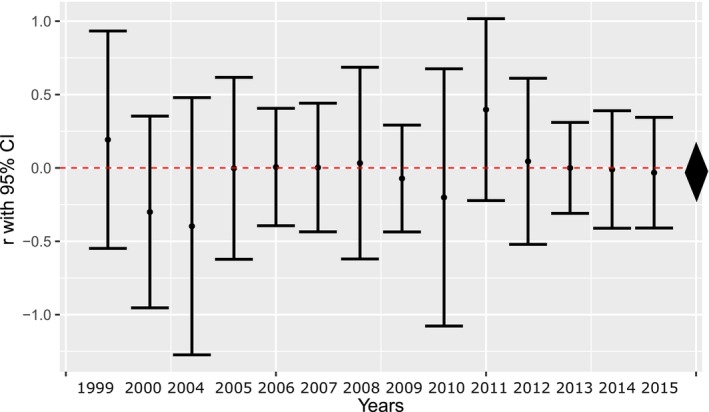
Yearly Mantel correlations (*r*) of repertoire similarity and spatial proximity (95% confidence intervals were calculated based on the variance of the correlations transformed to Fisher's *z*). The average effect size and its 95% credible interval are depicted as a diamond on the right side of the figure.

## 4 Discussion

In this field study, we found that some elements of the song performance were associated with the position of male birds in a social network. Specifically, we found that close‐collared flycatchers sang with different minimum frequencies and repertoire sizes. Regardless, the strength and direction of assortativity varied substantially over the years, and the song traits generally followed independent patterns. Social environmental and demographic parameters, such as the average number of detected displaying males or the proportion of immigrant and 1‐year‐old birds, were not related to the yearly assortativity estimates of song. We did not find age‐dependent settlement or spatial patterns in the repertoire similarity on a two‐kilometer scale. Our results of the generally negative assortment by the song traits suggest that collared flycatchers are unlikely to learn song elements from their neighbors or adjust their songs to be similar to their neighbors; instead, they may try to sing differently. Our results thus highlight that social network positions may shape communication in a territorial songbird regarding some song characteristics. However, within‐individual variability is high in the investigated song traits, except for repertoire size, and its inclusion resulted in broader confidence ranges around the assortativity estimates, which should be considered during the interpretation of our results.

Neighboring males sang songs with different minimum frequencies. Due to the correlative nature of our study, we cannot be sure whether different singing birds settled next to each other or whether they became different after territory establishment. Plastic adjustment is a more probable mechanism based on previous results of social environment‐dependent song plasticity in the study species (Jablonszky et al. 2021; Jablonszky et al. 2022b), and because song consistency was lower in collared flycatcher males that changed nest boxes within a year than in birds that remained at the same nest box between two recordings (Zsebők et al. 2017). Nevertheless, the pattern in minimum frequency (coupled with the similar trend in mean frequency) indicates that birds settle in a way or change their song to be more different in the neighborhood, maybe to remain audible and distinguishable. Importantly, this frequency difference did not seem to arise from different repertoire content in the neighboring birds, as we did not find spatial patterns in repertoire similarity. Interspecific studies repeatedly found frequency differences between the songs of bird species living in the same habitat (Luther 2009; Malavasi and Farina 2013; Hamao et al. 2016). Minimum frequency seems to be a flexible song trait that promotes audibility also at the within‐species level. For example, European blackbirds ( *Turdus merula* ) and oriental magpie robins ( *Copsychus saularis* ) adjusted the maximum and minimum frequency of their songs to noise levels (Mendes et al. 2011; Hanafi et al. 2019). Furthermore, the frequency of songs of great tits ( *Parus major* ; Bueno‐Enciso et al. 2016), fuscous honeyeaters ( *Lichenostomus fuscus* ; Goretskaia et al. 2018), satin bowerbirds ( *Ptilonorhynchus violaceus* ; Nicholls and Goldizen 2006), and Madagascar paradise flycatchers ( *Terpsiphone mutata* ; van Dongen and Mulder 2006) varied according to vegetation density that determines sound transmission.

The negative assortativity in repertoire size is more puzzling. Repertoire size may be connected to individual quality (Garamszegi et al. 2007; Hegyi et al. 2010), and birds of similar quality may avoid settling near each other. Having a neighbor with a lower‐quality song may be advantageous for the focal male if females make mating decisions based on the relative performance of males in the vicinity (Otter et al. 1999). Another possibility is that poor‐quality males may choose to settle down in the proximity of a male with a large repertoire size to have a higher chance to attract females (i.e., females are attracted by the neighbor male with a large repertoire size and this also increases the chance of pairing with the male with a low repertoire size). Accordingly, there is some evidence that birds settle down near and learn songs from more experienced and/or better quality neighbors in other species (Beecher et al. 1994; Ortega et al. 2014; Beecher 2017). This phenomenon is also similar to the leks or hidden leks (that can also occur in territorial birds) of various species, when subordinate individuals gather around high‐quality males (Höglund and Robertson 1990; Cockburn et al. 2009) and those closer to central, dominant males have higher breeding success (Fiske et al. 1998; Cockburn et al. 2009; Bro‐Jorgensen 2011). Settling near a better‐quality male may also have advantages in terms of territory defense, as chipping sparrows ( *Spizella passerina* ) form alliances (toward high‐quality opponents) preferentially with males of better vocal performance than themselves (Goodwin and Podos 2014). Similar mechanisms can also be an alternative explanation for the pattern found in minimum frequency, although its relationship with individual quality is less evident in our population (Garamszegi et al. 2004; Hegyi et al. 2010). However, according to our results, this potential quality‐dependent settlement is not connected to age. We cannot exclude that the patterns in song reflect genetic differences as relatives may settle further from each other (Wheelwright and Mauck 1998; Ishibashi and Saitoh 2008; Szulkin and Sheldon 2008). However, this explanation is not likely as the heritability of song is low in our study species (Jablonszky et al. 2022a) and generally in birds (Labra and Lampe 2018; Lewis et al. 2021).

It is important to acknowledge a limitation of our study: we sampled only approximately 19% of the population of the breeding males, so our results should be considered with caution. However, our rigorous field protocol allowed us to record most of the stable singing birds, whereas those singing less frequently (and possibly overlooked) might have less influence on the songs of others. Nevertheless, a larger sample size might have revealed stronger relationships among additional song traits.

Several studies investigated spatial patterns in bird songs on various spatial scales, but usually, they found more similar songs in neighborhoods (Briefer et al. 2008; Draganoiu et al. 2014; Quiroz‐Oliva and Sosa‐López 2022). This similarity between close birds was associated with increased reproductive success (Wegrzyn and Leniowski 2010; Poesel et al. 2012; Nelson and Poesel 2013) and lowered aggression from neighbors (Wilson and Vehrencamp 2001; Draganoiu et al. 2014). Regardless of the advantages of singing similar songs in neighborhoods, some exceptions are reported in rufous‐collared sparrows ( *Zonotrichia capensis* ), where closer birds sang more dissimilar songs in the forest (Laiolo 2011), in white‐throated thrushes ( *Turdus assimilis* ; Vargas‐Castro 2015), and in nightingales ( *Luscinia megarhynchos* ; Hultsch and Todt 1981), where song sharing was higher with neighbors at an intermediate distance than with adjacent neighbors. Our results are also partly corroborated by a previous result from our population, as we found that repertoire overlap is positively related to the distance between pairs of collared flycatcher males (Garamszegi et al. 2012). Thus, close birds sang more differently in both studies, although we did not find spatial patterns in repertoire similarity. This difference may be explained by the variance between years, as the previous study included only 3 years, or by the different spatial scales (a few 100 m compared to the 2 km in this study). Our finding of a positive relationship between song similarity and distance in some song traits instead of the most widespread negative relationship may be explained by the biology of the study species. Collared flycatchers are long‐distance migrants and the population turnover is high (based on our ringing records 18% ± 3% of the breeding birds bred again in the next year regarding the consecutive study years), so the benefit of singing similarly to their neighbors may be low, as was suggested previously for other migratory birds (Kroodsma et al. 1999; Nelson et al. 2001; Yoon et al. 2013). Collared flycatchers usually sing for a few weeks at the beginning of the breeding season, when neighbors may change on a daily basis, so song similarity with these neighbors may have little advantage in promoting communication. Instead, it may have more benefits to advertise their quality and express individuality with individual‐specific songs. Although we lack data to make a strong conclusion, we assume that the mechanism of the observed song differences is both within‐individual plasticity [both minimum frequency and repertoire size were previously found to change with binary age (Garamszegi et al. 2007; Jablonszky et al. 2021)] and the settlement of immigrants in the area.

Yearly assortativity coefficients varied considerably over the years. Interestingly, regarding most of the song traits, these changes over the years were independent from those of the other song traits. Probably some environmental factors caused the high variation over the years, but we could not determine which one, as the average number of detected displaying males (estimating the number of males with which the focal male had to compete for nest holes and females), the proportion of immigrants, and the proportion of young birds in the population did not influence song similarity between neighbors. However, it should be noted that the moderator variables were estimated with some noise (e.g., we probably could not locate all displaying males; some unringed birds classified as immigrants may were born in natural nest holes in our study area). Also, our sample size for the analysis corresponded to the number of study years (*N* = 19), so the lack of relationships should be considered cautiously. Other factors not investigated in this study, like abiotic factors, could also drive the variability over years. Nevertheless, the high among‐year variability in the assortativity coefficient estimates indicates that not a single hypothesis explains our results. Instead, multiple mechanisms and environmental factors may interactively shape the pattern of associations between the birds; for example, the intensity of competition may affect them differently in good and poor years.

In summary, we found differences in some song traits of a wild passerine according to the individuals' position in a social network. Specifically, regarding minimum frequency and repertoire size, close birds settled or changed their songs in a way to be more different from each other. The observed patterns were consistent with a hypothesis of competition as a mechanism. Still, the exact factors causing the substantial variance among years in assortativity should be investigated further. Overall, our results highlight the importance of investigating traits playing a role in communication together with the underlying social networks due to their complex interactions.

## Author Contributions

**Mónika Jablonszky:** conceptualization (supporting), formal analysis (lead), investigation (equal), visualization (lead), writing – original draft (lead). **Miklós Laczi:** investigation (equal), writing – review and editing (equal). **Gergely Nagy:** investigation (equal), writing – review and editing (equal). **Zoltán Tóth:** formal analysis (supporting), methodology (supporting), visualization (supporting), writing – review and editing (equal). **Sándor Zsebők:** conceptualization (equal), data curation (lead), formal analysis (supporting), investigation (equal), methodology (equal), software (equal), writing – review and editing (equal). **László Zsolt Garamszegi:** conceptualization (equal), funding acquisition (lead), investigation (equal), writing – review and editing (equal).

## Conflicts of Interest

The authors declare no conflicts of interest.

## Supporting information


Data S1.

## Data Availability

All data and code used for the analyses is deposited in Figshare: https://doi.org/10.6084/m9.figshare.28416929.
