# Supplementary material for: Close Males Sing With Dissimilar Minimum Frequency and Repertoire Size in a Wild Passerine
Source: Ecol Evol. 2025 Apr 15;15(4):e71044. doi: 10.1002/ece3.71044 (PMC11997371; doi:10.1002/ece3.71044)
Supplement: Supplementary file 1 — Data S1. [file ECE3-15-e71044-s001.docx]

Supplementary material for: **Close males sing with dissimilar minimum frequency and repertoire size in a wild passerine**

Table S1: Yearly sample sizes for the recorded birds. In the first row sample sizes for the song traits and in the second row sample sizes for age (as data on age was missing for some birds) are shown

|  | 1999 | 2000 | 2004 | 2005 | 2006 | 2007 | 2008 | 2009 | 2010 | 2011 | 2012 | 2013 | 2014 | 2015 | 2016 | 2017 | 2018 | 2019 | 2020 |
| --- | --- | --- | --- | --- | --- | --- | --- | --- | --- | --- | --- | --- | --- | --- | --- | --- | --- | --- | --- |
| song traits | 10 | 12 | 8 | 13 | 27 | 23 | 12 | 32 | 8 | 13 | 15 | 43 | 27 | 30 | 45 | 24 | 27 | 23 | 29 |
| age | 10 | 12 | 8 | 13 | 27 | 17 | 8 | 31 | - | - | - | 27 | 20 | 24 | 33 | - | 19 | 19 | - |

Fig S1: Example of two different syllable types a) and b), with multiple syllables shown from multiple individuals


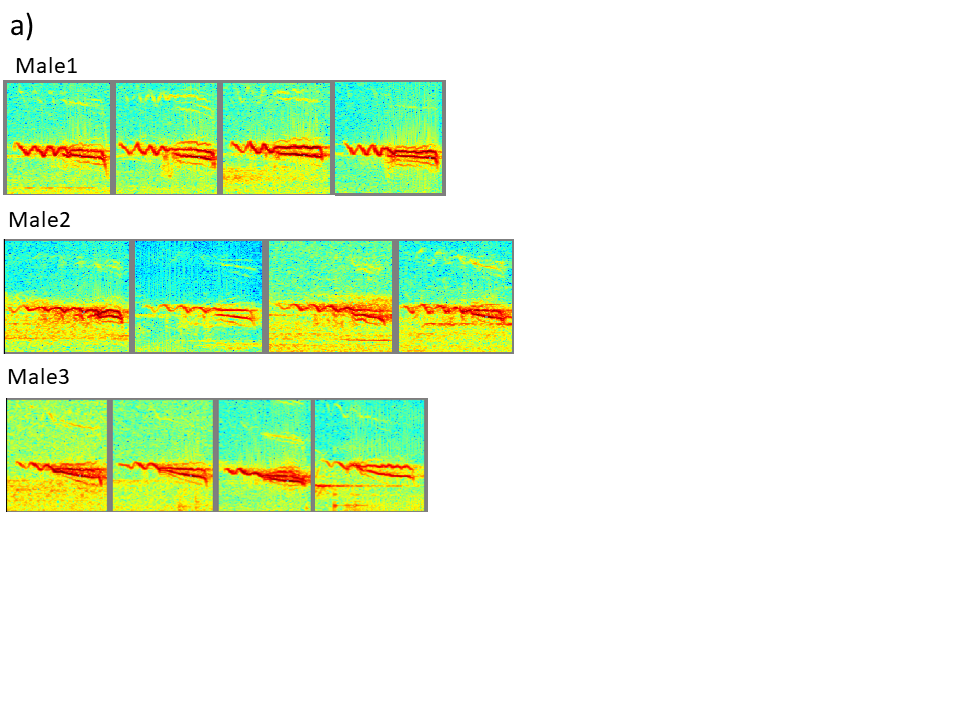

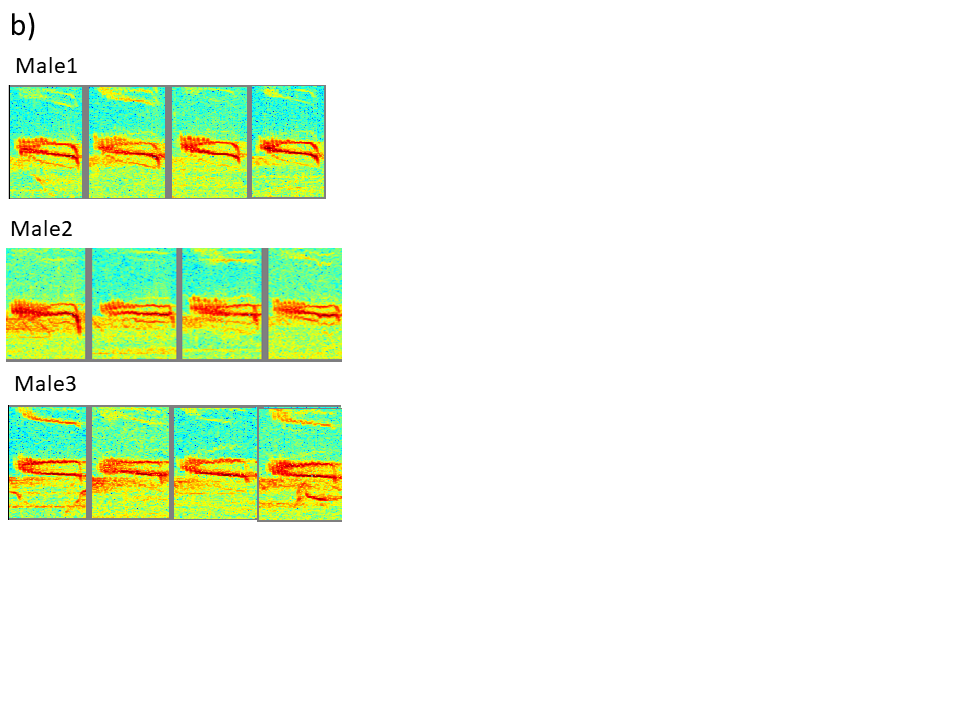


Table S2: Summary statistics for the investigated song traits

|  | Mean | Standard deviation | Range |
| --- | --- | --- | --- |
| Mean frequency (Hz) | 5477 | 269.237 | 4426-6405 |
| Minimum frequency (Hz) | 3346 | 482.425 | 2430-4981 |
| Maximum frequency (Hz) | 7245 | 344.190 | 6261-9377 |
| Song length (s) | 2.833 | 0.747 | 1.461-6.035 |
| Tempo (1/s) | 3.553 | 0.313 | 2.242-4.569 |
| Complexity | 0.733 | 0.085 | 0.450-0.929 |
| Repertoire size | 42.340 | 19.323 | 1-127 |

Text S1.

Because the repeatability of song characteristics is low and we could not consider the within-individual variance during the calculation of weighted assortativity coefficients, we carried out multiple additional analyses. These analyses excluded repertoire size, for which we only have one value per individual.

We calculated within-recording repeatability for the song characteristics including songs based on which the means used in the main text were calculated (15 songs per individual). We used ‘rptGuassian’ function from the ‘rpt’ package ([Stoffel et al. 2017](#_ENREF_2)) and inserted the recording ID as a random factor. We excluded here the birds that had two recordings from different years as, due to the limited sample size (N=12), we could not reliably separate the variance for this level. We found that the repeatability was between 0.175 and 0.270 and was always significant (the p-value of the likelihood ratio test was <0.001 in all cases; Table S3). These values align well with our previous results on within-day repeatability ([Zsebők et al. 2017](#_ENREF_4)).

Table S3: Within-recording repeatability values for the six song traits with 95% confidence intervals (CI)

|  | Mean frequency | Minimum frequency | Maximum frequency | Song length | Tempo | Complexity |
| --- | --- | --- | --- | --- | --- | --- |
| Repeatability (95% CI) | 0.270 (0.235, 0.300) | 0.227 (0.197, 0.257) | 0.175 (0.148, 0.205) | 0.193 (0.162, 0.221) | 0.255 (0.224, 0.290) | 0.197 (0.167, 0.225) |

Next, we calculated the weighted assortativity coefficients for the six song traits 100 times choosing one song per individual randomly. We otherwise used the same method as described in the main text.

We run linear mixed models considering the within-individual variance between the songs using ‘brm’ function from the ‘brms’ package ([Bürkner 2018](#_ENREF_1)) that allowed us to include the variability of the response variable in the model. We calculated this variability based on a modified equation for the unconditional variance during full-model averaging ([Symonds & Moussalli 2011](#_ENREF_3)), using equal weights (0.01) for all of the 100 assortativity coefficients estimated based on randomly chosen songs. Otherwise, the structure of the models was similar to the ones in the main text, including a correlation matrix reflecting the expected among-year similarity. We found that the estimates for average effect size were qualitatively similar to that obtained with our original analysis, but neither of them was significant (Table S4, Fig S2). This could be attributed to the data-hungriness of the methods which was reflected also in the bad diagnostics of the models (namely posterior predictive checks, comparing observed data to simulated data from the posterior predictive distribution, Fig S3). So these results should be considered with caution.

Table S4: Average effect sizes for the weighted assortativity coefficients of the six song traits considering also within-recording variation

|  | Mean frequency | Minimum frequency | Maximum frequency | Song length | Tempo | Complexity |
| --- | --- | --- | --- | --- | --- | --- |
| Average effect size (95% CI) | -0.16  (-0.34, 0.03) | -0.14  (-0.32, 0.05) | -0.11  (-0.30, 0.07) | -0.13  (-0.32, 0.06) | -0.12  (-0.31, 0.08) | -0.13  (-0.33, 0.07) |

Fig S2: Mean yearly assortativity coefficient estimates (*r*) for all investigated song traits with respective 95% credible intervals based on the 100 runs with random songs. The average effect size and its 95% credible interval from the Bayesian linear mixed models is depicted as a diamond on the right side for each song trait


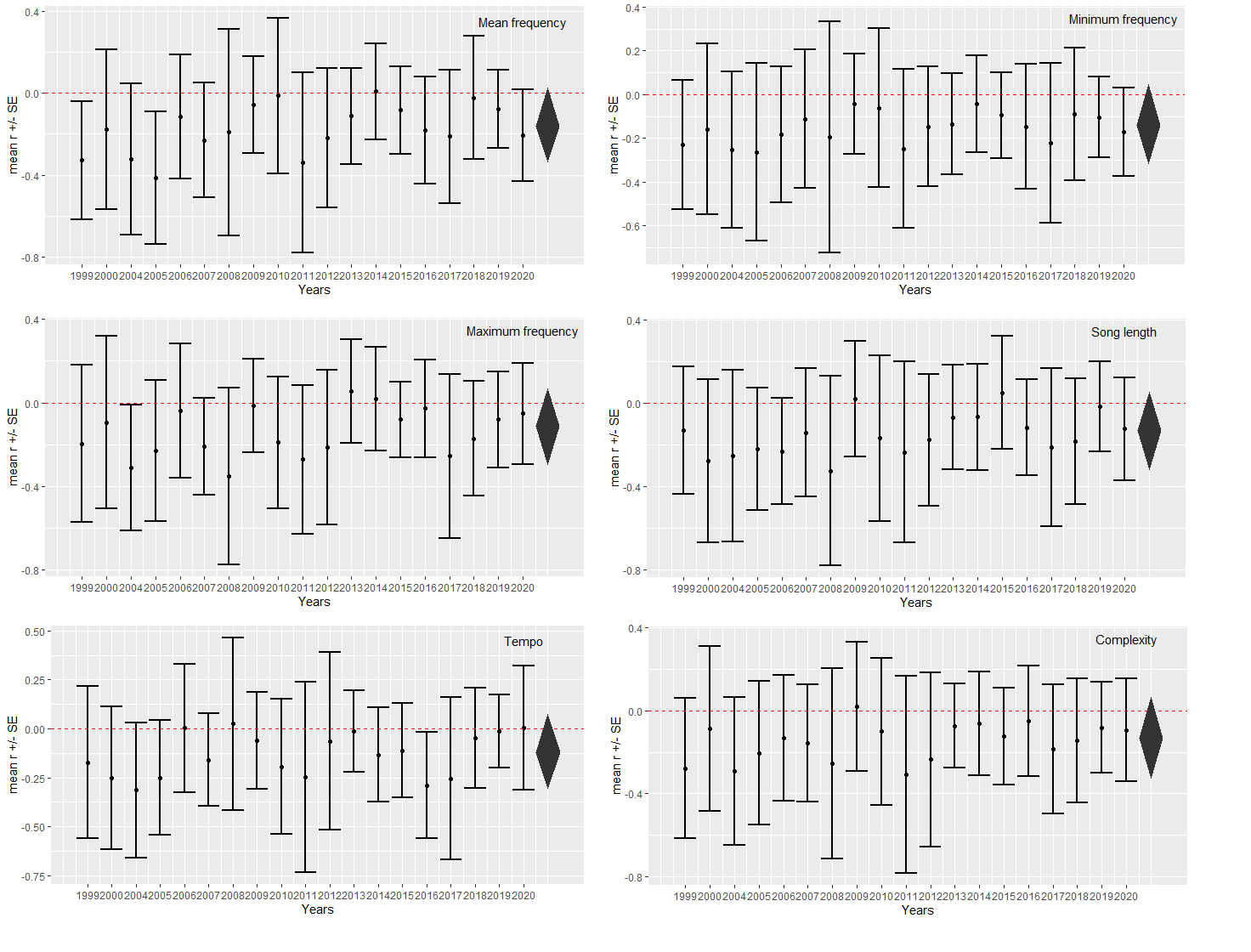


Fig S3: Posterior predictive check of the model with mean frequency as a response variable. *y* depicts observed data and *y_rep_* depicts simulated data from the posterior predictive distribution


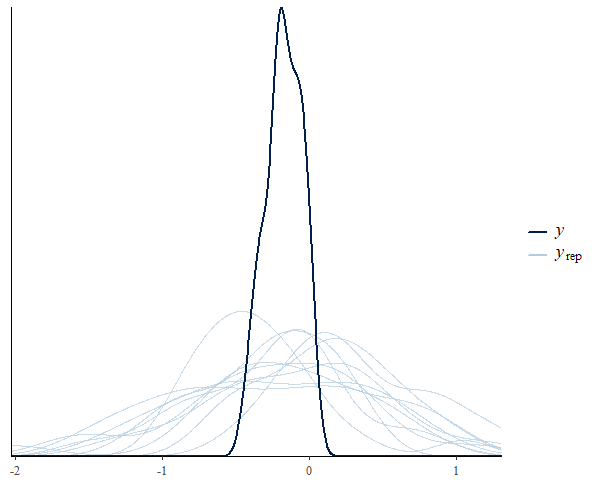


Density

Outcome

References

Bürkner P-C. 2018. Advanced Bayesian multilevel modeling with the R package brms. The R Journal 10:395-411. https://doi.org/10.32614/RJ-2018-017

Stoffel MA, Nakagawa S, Schielzeth H. 2017. rptR: repeatability estimation and variance decomposition by generalized linear mixed-effects models. Methods Ecol Evol 8:1639-1644. https://doi.org/doi:10.1111/2041-210X.12797

Symonds MRE, Moussalli A. 2011. A brief guide to model selection, multimodel inference and model averaging in behavioural ecology using Akaike's information criterion. Behav Ecol Sociobiol 65:13-21. https://doi.org/10.1007/s00265-010-1037-6

Zsebők S, Herczeg G, Blázi G, Laczi M, Nagy G, Szász E, Markó G, Török J, Garamszegi LZ. 2017. Short- and long-term repeatability and pseudo-repeatability of bird song: sensitivity of signals to varying environments. Behav Ecol Sociobiol 71:154. https://doi.org/10.1007/s00265-017-2379-0
